# The dynamic functional connectivity peak index: Detection of interictal epileptic activity with fMRI

**DOI:** 10.1002/epi.70335

**Published:** 2026-06-11

**Authors:** Lucas E. Sainburg, Alexandra Roche, Ghassan S. Makhoul, Baxter P. Rogers, Shawniqua Williams Roberson, Stefano Meletti, Anna E. Vaudano, Catie Chang, Dario J. Englot, Victoria L. Morgan

**Affiliations:** ^1^ Department of Biomedical Engineering Vanderbilt University Nashville Tennessee USA; ^2^ Vanderbilt University Institute of Imaging Science Vanderbilt University Medical Center Nashville Tennessee USA; ^3^ Department of Neurology Vanderbilt University Medical Center Nashville Tennessee USA; ^4^ Department of Biomedical, Metabolic and Neural Science University of Modena and Reggio Emilia Modena Italy; ^5^ Neurophysiology Unit and Epilepsy Center, Neuroscience Department Modena AOU Modena Italy; ^6^ Department of Electrical and Computer Engineering Vanderbilt University Nashville Tennessee USA; ^7^ Department of Neurological Surgery Vanderbilt University Medical Center Nashville Tennessee USA

**Keywords:** dynamic functional connectivity, epilepsy surgery, fMRI, interictal, temporal lobe epilepsy

## Abstract

**Objective:**

Epileptogenic zone (EZ) localization is crucial for surgical treatment of patients with medication‐resistant epilepsy. Although simultaneous electroencephalography (EEG) and functional magnetic resonance imaging (fMRI) can detect interictal discharges for EZ localization, clinical adoption is limited by the need for specialized equipment and expertise. Prior EEG‐fMRI work has shown that interictal discharges produce fMRI activation in the EZ and deactivation in the default mode network. Here we use dynamic functional connectivity (dFC) to detect these opposing activations and localize potential interictal activity using fMRI without simultaneous EEG in a cohort composed primarily of temporal lobe epilepsy (TLE).

**Methods:**

We quantified dFC with the edge timeseries approach and present examples of negative dynamic functional connectivity peaks following interictal discharges in two patients with TLE who received simultaneous EEG‐fMRI. We defined the rate of these peaks as the “dFC peak index,” hypothesizing it to be elevated in epileptic tissue. We assessed this hypothesis in 62 medication‐resistant patients with focal epilepsy who underwent fMRI *without* simultaneous EEG (49 unilateral temporal, 11 bilateral temporal, 1 frontal, and 1 parietal). We Z‐scored dFC peak index in each patient to healthy controls (*n* = 109) to identify regions with abnormally high values and tested whether these aligned with patients' EZs or correlated with clinical parameters.

**Results:**

The dFC peak index was elevated in epileptic medial temporal structures in patients with TLE (*p*_FWE_ <.05). Resection of regions with higher values correlated with better seizure outcomes after surgery (area under the curve [AUC] = .80, *p* = .0002), even in patients without hippocampal lesions on MRI (AUC = .79, *p* = .04).

**Significance:**

This study presents the dFC peak index, which aims to detect interictal epileptic activity using fMRI without simultaneous EEG. Our results indicate that this measure is elevated in epileptic tissue in TLE, suggesting that it could provide unique information to guide epilepsy surgery.

**Key points**

- We developed the dynamic functional connectivity (dFC) peak index to detect potential interictal epileptic activity from functional magnetic resonance imaging (fMRI) without simultaneous electroencephalography (EEG).
- dFC peak index was elevated in the medial temporal lobes of patients with temporal lobe epilepsy, consistent with their epileptogenic zones.
- Hippocampal dFC peak index laterality distinguished right from left temporal lobe epilepsy.
- Resection of high dFC peak index corresponded to better seizure outcomes, even in patients without hippocampal lesions on MRI.
- The dFC peak index may provide unique information to support localization and surgical planning.

## 1 INTRODUCTION

Localization of the epileptogenic zone (EZ) is crucial for effective surgical treatment in patients with focal epilepsy. EZ localization can guide either surgical resection of the EZ or neurostimulation device selection and electrode placement in patients who are not candidates for resection.^1^ There are several neuroimaging and electrophysiological techniques currently used to localize the EZ in the clinic.^2^, ^3^ However, ~38% of patients are still not seizure‐free after resection,^4^ in part due to mis‐localization of the EZ.^5^

EZ localization is carried out by detecting anatomic and physiological characteristics of epileptic tissue, including abnormalities in brain structure, interictal hypometabolism, electrophysiological seizure onset, and interictal epileptic discharges.^2^, ^3^ Interictal discharges can be coarsely localized with scalp electroencephalography (EEG), but this localization can be improved by combining EEG with simultaneous functional magnetic resonance imaging (fMRI) to detect the fMRI signal increases in the tissue generating the discharges.^6^, ^7^ However, clinical utilization of simultaneous EEG‐fMRI in epilepsy has not been widely adopted due to the need for MRI‐compatible EEG equipment as well as specialized expertise in the collection and analysis of the data. Moreover, EEG‐fMRI cannot be carried out in patients who have interictal discharges that are very infrequent or undetectable on scalp EEG.^8^, ^9^

Although EEG‐fMRI studies of interictal discharges are typically aimed at localizing fMRI signal increases in the EZ, several studies have also found fMRI signal decreases in response to discharges in the default mode network (DMN),^10^, ^11^, ^12^, ^13^ a set of cortical regions that are active at rest and implicated in self‐reference and memory.^14^ Fahoum and colleagues have additionally found intracranial electrophysiological correlates of these fMRI signal decreases in the DMN^15^ similar to the correlates of fMRI decreases seen during seizures,^16^ supporting that discharges may suppress neural activity in this network. This DMN suppression has been suggested as a potential mechanism for momentary lapses in consciousness and attention during interictal discharges.^12^, ^15^

In this study, we aimed to detect interictal epileptic activity with fMRI without simultaneous EEG. Prior work has attempted this by detecting significant fMRI signal increases in the EZ^17^, ^18^, ^19^; however, these signal increases can be difficult to distinguish from normal resting oscillatory activity in the brain. Here we aimed to improve the specificity of interictal epileptic activity detection by isolating timepoints at which the fMRI signal in a region of interest increases (activates) *and* the fMRI signal in the DMN decreases (deactivates), based on prior simultaneous EEG‐fMRI literature.^10^, ^11^, ^12^, ^13^ These opposing signal changes lead to transient negative dynamic functional connectivity (dFC)^20^ between these regions that we can detect with dFC analyses.

We first present examples of transient negative peaks in dFC following interictal discharges in two patients with mesial and lateral temporal lobe epilepsy (TLE) and simultaneous EEG‐fMRI. We then termed the rate of these negative dFC peaks the “dFC peak index” and tested whether increased dFC peak index, as compared to healthy controls, coincided with the laterality and location of the EZ in a cohort of patients with focal epilepsy who underwent fMRI *without* simultaneous EEG. This cohort was comprised primarily of patients with mesial TLE, to serve as a well‐studied and relatively homogeneous model of focal epilepsy, as well as two patients with frontal and parietal lobe epilepsy, included for exploratory analysis. Finally, we assessed whether dFC peak index was related to other clinical measures that may be associated with interictal epileptic discharges. Overall, the goal of this study was to detect interictal epileptic activity using fMRI without simultaneous EEG, which may have potential as a non‐invasive method to aid in guidance of epilepsy surgery.

## 2 METHODS

This Methods section is split into two parts. Part 1 describes the concept of negative dFC peaks using examples of EEG‐fMRI data in two patients. Part 2 describes the methods used for the primary analysis of the study, that is, the assessment of the dFC peak index, in a large cohort of patients who underwent fMRI without simultaneous EEG. The following Results section is based solely on Part 2 of the Methods section.

### 2.1 Part 1: Negative dFC peaks and examples in EEG‐fMRI data

This section includes examples of negative dFC peaks in two patients with simultaneous EEG‐fMRI. The details on these data and methods are included in the Supporting Information.

#### 2.1.1 Calculation of negative dFC peaks

**FIGURE 1:**
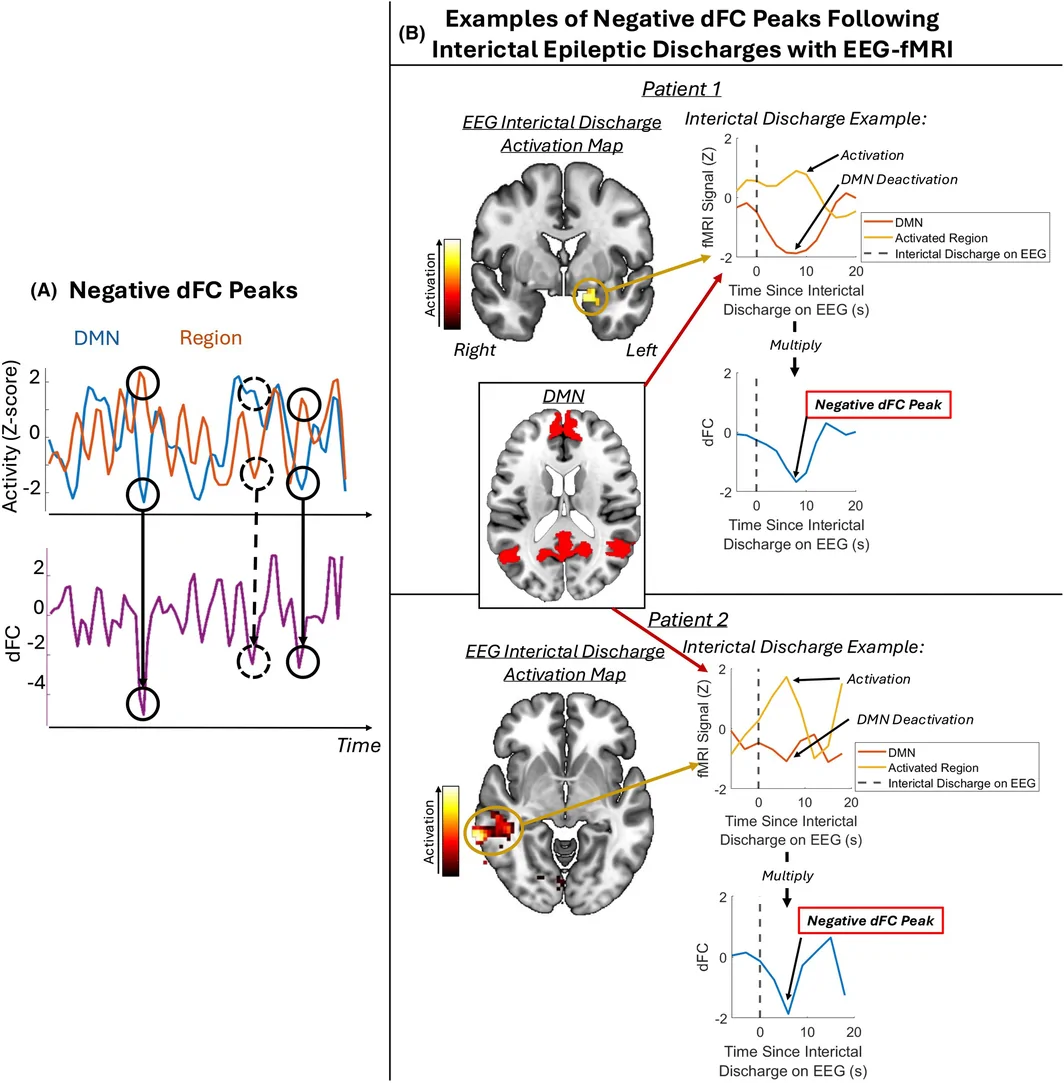
Negative dFC peaks. (A) Description of negative dFC peaks. Temporally Z‐scored timeseries for the DMN and a region are shown. The two timeseries are multiplied together at each timepoint to calculate the dFC timeseries between the two regions. Negative dFC peaks are highlighted with black circles, with solid circles denoting peaks of interest (DMN deactivation) and dotted circles denoting peaks of no interest (DMN activation). (B) Examples of negative dFC peaks following interictal epileptic discharges in a patient with left medial temporal lobe epilepsy (top) and right lateral temporal lobe epilepsy (bottom). The EEG‐based interictal epileptic discharge activation maps are shown on the left along with a map of the DMN. The timeseries for the DMN, the activated region from the interictal discharges, and the dFC between the two regions are shown in red, yellow, and blue, respectively. DMN, default mode network; dFC, dynamic functional connectivity; EEG: electroencephalography; fMRI, functional magnetic resonance imaging.

We calculated dFC between pairs of regions using the edge timeseries method^21^ as follows: The fMRI timeseries from two regions were first de‐meaned and divided by their temporal standard deviations (SD; i.e., temporally Z‐scoring each timeseries) and were then multiplied together at each timepoint to calculate dFC (Figure 1A). A negative peak in the dFC timeseries arises at any timepoint at which one region's signal increases and the other region's signal decreases (Figure 1A, circles). We are specifically interested in negative dFC peaks highlighted by the solid circles in Figure 1A, at which the signal in the DMN decreases and the signal in the region of interest increases. We are not interested in the opposite type of negative dFC peak at which the DMN signal increases, as highlighted by the dotted circles in Figure 1A.

#### 2.1.2 Relating negative dFC peaks to EEG‐fMRI

Using EEG‐fMRI, we show examples of negative dFC peaks following interictal epileptic discharges in two patients with mesial and lateral TLE. EEG‐fMRI data were acquired at the University of Modena and Reggio Emilia (acquisition and processing details in Supporting Information) and interictal epileptic discharges were marked on the preprocessed EEG recordings by an experienced electroencephalographer (A.E.V.). fMRI activation maps of the interictal epileptic discharges were created with a general linear model. The timeseries from the DMN (see Segmentation below for details), the activated region from the interictal discharges, and the dFC timeseries between these two regions were calculated.

Regions of activation were found in the left medial temporal and right lateral temporal lobes of Patients 1 and 2, respectively (Figure 1B). In both patients, the DMN deactivates simultaneously with the activation in the temporal lobe region, ~6–8 s after the interictal discharge on EEG. This results in a negative peak in dFC between these regions (Figure 1B).

### 2.2 Part 2: Assessment of the dFC peak index in fMRI data without EEG (primary analysis)

#### 2.2.1 Participants

This study included 62 patients with medication‐resistant focal epilepsy who underwent surgical treatment for their seizures between 2012 and 2024 and had fMRI data *without* simultaneous EEG collected. This cohort consisted of 49 patients with mesial unilateral TLE (16 left sided), 11 patients with bilateral TLE (for laterality analysis), and two patients who underwent resections in their frontal and parietal lobes. Patients with unilateral TLE underwent either a selective amygdalohippocampectomy (SAH; *n* = 38), an anterior temporal lobectomy (ATL; *n* = 10), or a laser ablation of the amygdala and hippocampus (*n* = 1), and seizure outcomes were recorded 3 years after surgery on the Engel scale.^22^ Patients with bilateral TLE were diagnosed based on bilateral ictal activity on scalp and/or intracranial EEG and were subsequently treated with neurostimulation. All patients received standard assessments prior to surgery, including scalp EEG, structural MRI, interictal fluorodeoxyglucose–positron emission tomography (FDG‐PET), and neuropsychological testing, and underwent treatment at Vanderbilt University Medical Center after careful review of these data by a multidisciplinary epilepsy surgery team. A group of 109 healthy controls were included as a normal reference. This study was approved by the Vanderbilt University Institutional Review Board, and informed consent was obtained from all participants.

#### 2.2.2 Preoperative fMRI acquisition and preprocessing

MRI scanning was carried out on a Philips (Best, Netherlands) 3 T MRI using a 32‐channel head coil. All participants underwent a scanning session that included a T1‐weighted three‐dimensional (3D) scan for anatomy (1 mm isotropic), a T1‐weighted 2D scan in the same slice orientation as the functional images for registration (1 × 1 × 3.5 mm with a .5 mm gap), and 20 min of T2*‐weighted eyes‐closed resting‐state fMRI (3 × 3 × 3.5 mm with a .5 mm gap, repetition time = 2 s, echo time = 35 ms). A pulse oximeter and respiratory belt collected cardiac and respiratory fluctuations during fMRI scans for preprocessing.

fMRI data were corrected for physiological noise with retrospective image correction (RETROICOR)^23^ using the cardiac and respiratory signals. This was followed by preprocessing with Statistical Parametric Mapping 12 (SPM12; https://www.fil.ion.ucl.ac.uk/spm/software/spm12/) that included slice timing correction, motion correction, normalization to Montreal Neurological Institute (MNI) coordinates, and spatial smoothing with a 6 mm full width at half‐maximum Gaussian kernel. Next, the mean white matter and cerebrospinal fluid signals, along with the six translational and rotational motion parameters, were regressed out of the fMRI timeseries. The data were then bandpass filtered between .0083 to .1 Hz.

#### 2.2.3 Segmentation

Presurgical T1‐weighted scans were segmented into cortical and subcortical regions using a multi‐atlas algorithm,^24^ and hippocampal subfields were segmented with FreeSurfer 6^25^ and merged into anterior and posterior hippocampi^26^ bilaterally for a total of 117 regions. The hippocampi were split into anterior and posterior segments, since SAH and ATL procedures often resect the anterior but not posterior portion of the hippocampus. The DMN was defined as the bilateral posterior cingulate cortex, precuneus, angular gyrus, and medial frontal cortex.^14^

All patients who received resections had a postoperative T1‐weighted MRI (1 mm isotropic) collected to delineate their resection cavity. ResectVol^27^ and SPM12 were used to segment resection cavities from the postsurgical T1‐weighted scans and register them to the presurgical scan. The percentage of each region resected was determined by applying the resection cavity to the presurgical atlas of regions. Resected regions were identified by having at least a 25% reduction in volume, similar to prior studies.^28^, ^29^ All resection cavities and regions were manually inspected and edited as needed.

#### 2.2.4 dFC peak index calculation

##### dFC Peak identification

**FIGURE 2:**
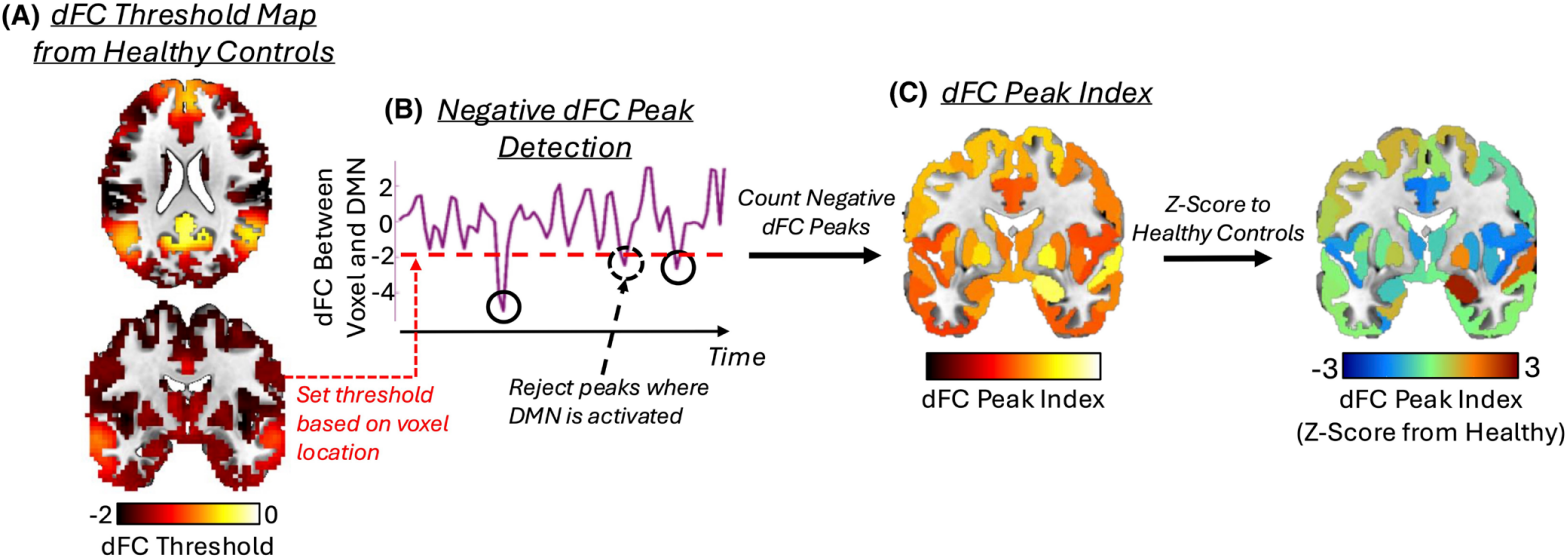
Calculation of patient dFC peak index maps. A voxel‐wise dFC threshold map derived from healthy controls (A) was used to set a threshold for negative dFC peak detection (B) based on voxel location. Peaks at which the DMN was activated were rejected and all the peaks at which the DMN was deactivated were counted and averaged within each region to obtain the regional dFC peak index map (C). dFC peak index was then Z‐scored to healthy controls at each region. DMN, default mode network; dFC, dynamic functional connectivity.

To detect negative dFC peaks between the DMN and other regions of the brain, the dFC timeseries was first calculated between each voxel and the DMN as described in Part 1 of the Methods (Figure 1A). Negative dFC peaks between the DMN and each voxel were detected (Figure 2B) as timepoints that fulfilled three criteria: (1) dFC at the timepoint was lower than the immediately preceding and following timepoints (negative peak); (2) the DMN signal was negative (deactivated) and the voxel signal was positive (activated); and (3) the dFC value was significantly negative, that is, less than a voxel‐specific dFC threshold (*p* < .05) determined from a null model in healthy controls (Figure 2A; see next paragraph for details).

Definition of significant decreases in the dFC timeseries were set at the fifth percentile (*p* = .05) for each control subject for each voxel. Then the median of these values across all healthy controls was taken as the group‐level *p* = .05 threshold at each voxel to produce the voxel‐wise dFC threshold map (Figure 2A). This threshold is used to identify significantly negative peaks in dFC timeseries (Figure 2B, red dotted line).

##### dFC peak index

The number of negative dFC peaks were then counted and averaged across voxels within each region to obtain the regional dFC peak index. Finally, the dFC peak index was Z‐scored to healthy controls at each region (Figure 2C).

#### 2.2.5 Analyses

##### Participant information

Age and sex were compared across patients with unilateral TLE, patients with bilateral TLE, and healthy controls. Each of the variables included in Table 1 were tested between Engel class I and Engel II–IV patients with unilateral TLE.

**TABLE 1:** Demographic and clinical information of patients with temporal lobe epilepsy and healthy controls.

	Unilateral TLE (*n* = 49)	Bilateral TLE (*n* = 11)	Healthy controls (*n* = 109)	*p*
Age, years	41.1 ± 12.0	38.4 ± 14.5	37.7 ± 13.4	.22^a^
Sex, % female	57.1	81.8	51.3	.14^b^
Duration of epilepsy, years	20.7 ± 15.0	14.9 ± 5.9		
Age at epilepsy onset, years	20.3 ± 15.2	23.6 ± 11.9		
HS on MRI, %	63.3	27.3		
Hypometabolism on FDG‐PET, %	75.5	40.0^1^		
Intracranial EEG testing, %	20.4	72.7		
Interictal EEG lateralization, %^2^	69.4	27.3		
Side of surgery, % Right^3^	67.3			
**Surgery type, %**				
SAH	77.6			
ATL	20.4			
SLAH	2.0			
**Postoperative pathology, %**				
HS	83.7			
Gliosis only	12.2			
Normal	2.0			
No pathology	2.0			
**3‐Year Engel outcome, %**				
I	59.2			
II	22.5			
III	12.2			
IV	6.1			

*Note:* ^1^One patient with bilateral TLE did not undergo an FDG‐PET scan. ^2^Interictal EEG lateralization was defined as presence of more interictal epileptic activity in one hemisphere compared to the other. ^3^None of the patients with bilateral TLE underwent a surgical resection. Continuous variables are reported as mean ± standard deviation and binary variables are reported as percentages.

Abbreviations: ATL, anterior temporal lobectomy; EEG, electroencephalography; FDG‐PET, fluorodeoxyglucose‐positron emission tomography; HS, hippocampal sclerosis; MRI, magnetic resonance imaging; SAH, selective amygdalohippocampectomy; SLAH, stereotactic laser amygdalohippocampectomy; TLE, temporal lobe epilepsy.

^a^ Kruskal–Wallis test.

^b^ Chi‐square test.

##### Regional group differences in dFC peak index

Regional group differences in dFC peak index between the patients with unilateral TLE and healthy controls were tested using dFC peak index values prior to Z‐scoring to controls, since the same healthy controls tested were used in the Z‐score procedure. The region labels of the patients with left TLE were flipped left–right prior to the test to orient regions as ipsilateral to the EZ. The region labels of an equal proportion of healthy controls (36 controls, ~33%) were also flipped left–right to reduce any bias introduced from flipping the regions of patients with left TLE.

##### dFC peak index laterality

dFC peak index laterality was calculated in the hippocampus by taking the dFC peak index of the right hippocampus minus the dFC peak index of the left hippocampus. Differences in dFC peak index laterality were tested between right TLE, left TLE, bilateral TLE, and controls. The performance of this single laterality measure to distinguish left from right TLE was determined using area under the receiver operating characteristic (ROC) curve (AUC) analysis across the whole cohort of patients with unilateral TLE as well as specifically in patients who did not have hippocampal sclerosis (HS) on MRI.

##### Comparison of resected dFC peak index to seizure outcomes after surgery

dFC peak index in the resection was compared between patients with unilateral TLE who had Engel I vs Engel II–IV seizure outcomes both at 1 and 3 years after surgery. The ability of the single measure of dFC peak index in the resection to distinguish patients with Engel I from Engel II–IV outcomes was determined with AUC analysis across the whole cohort of patients with unilateral TLE, as well as specifically in patients who were HS negative on MRI.

##### Clinical correlates of dFC peak index

Scalp EEG interictal epileptic discharge frequency was obtained from inpatient reports based on manual review of the EEG recordings by an epileptologist using the American Clinical Neurophysiology Society standard^30^ Abundant: 1 per 10 s; Frequent: 1 per min and < 1 per 10 s; Occasional: 1 per hour and < 1 per min; Rare: < 1 per hour; and None: no interictal epileptic discharges. Scalp EEG interictal epileptic discharge frequency was compared to dFC peak index within the resection.

dFC peak index was additionally compared to age, sex, duration of disease, age at onset, days since last seizure, and presence of HS on pathology.

##### 2.2.5.1 Statistical analysis

For all analyses: Kruskal–Wallis tests were used to compare continuous variables across three or more groups and Wilcoxon rank‐sum tests were used for comparisons between two groups. Chi‐square tests were used for comparisons of binary variables between groups. Associations between continuous variables were assessed using Spearman correlations. Bonferroni corrections were applied to control the family‐wise error rate for multiple comparisons.

## 3 RESULTS

### 3.1 Participant characteristics

Healthy controls, patients with unilateral TLE, and patients with bilateral TLE were not different in age or sex (*p* > .05, Table 1). In unilateral TLE, those with Engel I outcomes were more female (odds ratio [OR] 3.33, 95% confidence interval [CI] 1.01–10.97, *p* = .04) and had more localized hypometabolism on FDG‐PET (OR 4.17, 95% CI 1.04–16.62, *p* = .04) than patients with Engel II–IV outcomes, but no other clinical variables tested were related to outcomes in these patients (*p* > .05). The patients with frontal (18‐year‐old female) and parietal (28‐year‐old female) lobe epilepsy both had right hemispheric foci, localized hypometabolism on FDG‐PET, underwent intracranial EEG testing, had lateralizing interictal EEG activity, had Engel I outcomes 3 years after surgery, and were diagnosed with focal cortical dysplasia based on their postoperative pathology.

### 3.2 Regional group differences in dFC peak index between unilateral TLE and controls

Patients with unilateral TLE had increased dFC peak index in their ipsilateral anterior and posterior hippocampus, parahippocampal gyrus, and frontal pole compared to controls (*p*_FWE_ < .05; Figure S1). These patients had decreased dFC peak index in their ipsilateral medial precentral gyrus and contralateral parietal operculum compared to controls (*p*_FWE_ < .05; Figure S1).

### 3.3 Hippocampal dFC peak index laterality

**FIGURE 3:**
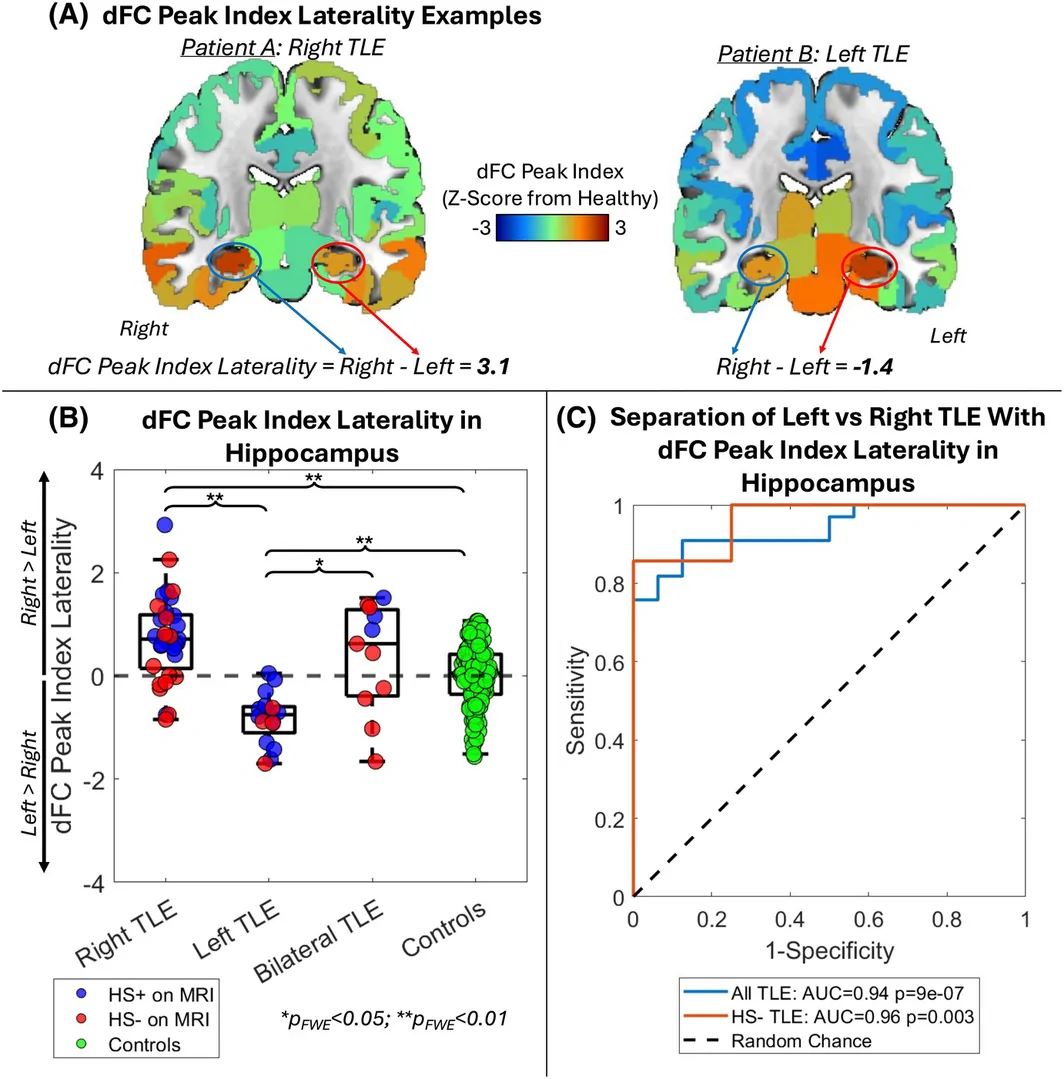
DFC peak index laterality of the hippocampus. (A) dFC peak index laterality calculations are shown for two example patients with right and left TLE. (B) dFC peak index laterality in the hippocampus is shown for patients with right TLE, left TLE, bilateral TLE, and healthy controls. Blue datapoints depict patients who were HS positive on MRI, red datapoints depict patients who were HS‐negative on MRI, and green datapoints depict healthy controls. The black dashed line represents no difference in dFC peak index between right and left hippocampi. (C) Separation of right vs left TLE with dFC peak index laterality in the hippocampus is shown using an ROC curve. The blue curve was calculated using all patients with unilateral TLE, whereas the orange curve was calculated using only patients who were negative for HS on their MRI. The black dashed line depicts random chance. dFC, dynamic functional connectivity; HS, hippocampal sclerosis; MRI, magnetic resonance imaging; TLE, temporal lobe epilepsy.

dFC peak index laterality was calculated in the hippocampus, with a positive number indicating higher dFC peak index in the right hippocampus and a negative number indicating higher dFC peak index in the left hippocampus. Examples of dFC peak index laterality calculations are shown in a patient with right TLE and a patient with left TLE in Figure 3A. Patients with right TLE had more right‐sided (higher) dFC peak index laterality than patients with left TLE (Cliff's *δ* .88, 95% CI .69–.96, *p*_FWE_ = 5 × 10^−6^; Figure 3B) and controls (Cliff's *δ* .50, 95% CI .23–.69, *p*_FWE_ = 7 × 10^−5^; Figure 3B). Patients with left TLE also had more left‐sided (lower) dFC peak index laterality than patients with bilateral TLE (Cliff's *δ* .64, 95% CI .09–.95, *p*_FWE_ = .04; Figure 3B) and controls (Cliff's δ .69, 95% CI .48–.82, *p*_FWE_ = 5 × 10^−5^; Figure 3B). dFC peak index laterality distinguished right from left TLE very well (AUC = .94, 95% CI .84–.98, *p* = 9 × 10^−7^; Figure 3C, Table S1), even in patients who were HS‐negative on their presurgical MRI (AUC = .96, 95% CI .64–1.00, *p* = .003; Figure 3C, Table S2).

### 3.4 dFC peak index relationship to seizure outcomes after surgery

**FIGURE 4:**
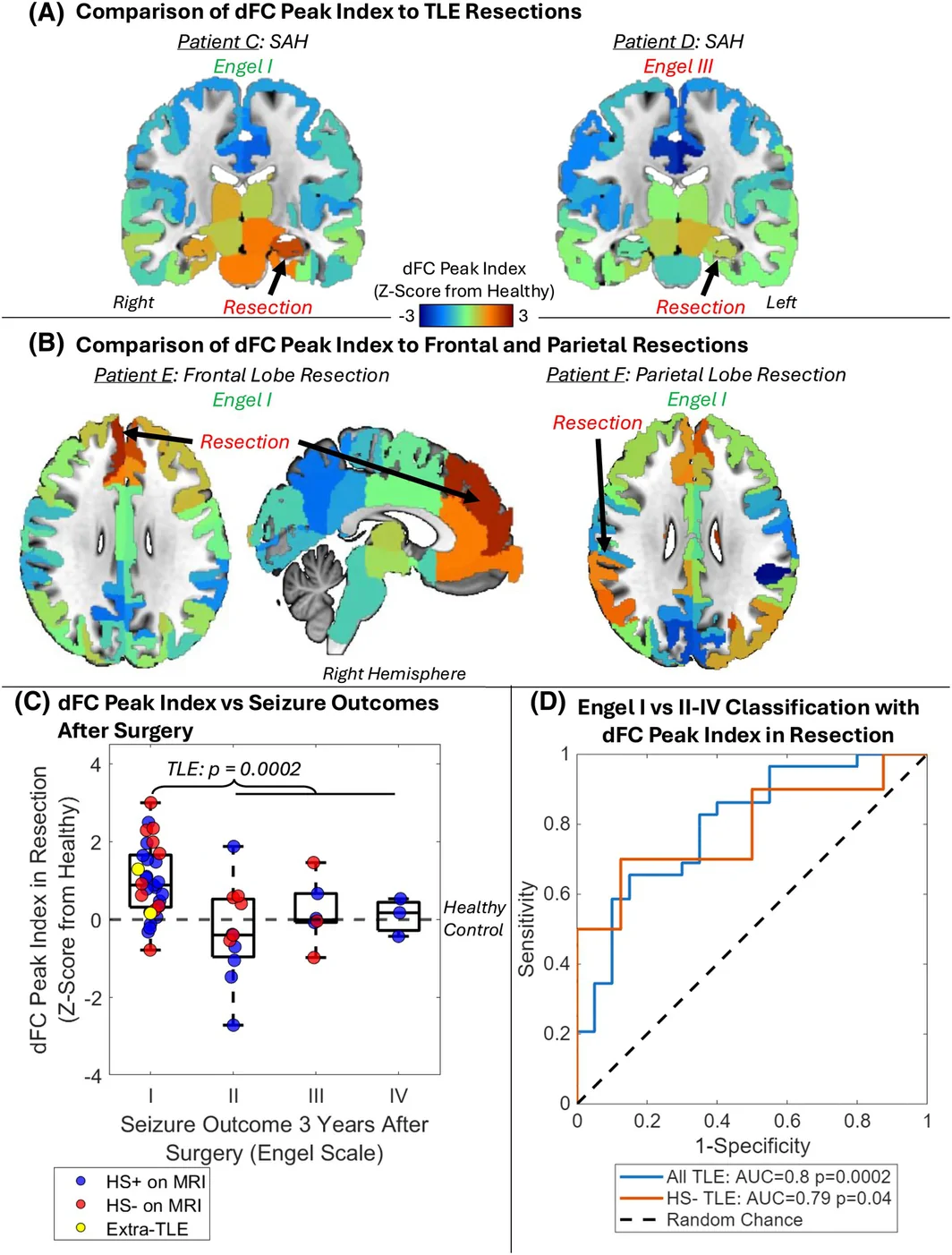
Surgical resection of high dFC peak index correlates with better 3‐year seizure outcomes. dFC peak index maps are shown in relation to resections of two patients with TLE who underwent SAH (A) and two patients who underwent resections in their frontal and parietal lobes (B). (C) dFC peak index in the resection vs seizure outcomes 3 years after surgery. Blue datapoints depict patients with TLE who were HS‐positive on MRI, red datapoints depict patients with TLE who were HS‐negative on MRI, and yellow datapoints depict the two patients with frontal and parietal lobe epilepsy (extra‐TLE). Note that the patients with frontal and parietal lobe epilepsy were not included in the statistics. The black dashed line represents the healthy control mean. (D) Separation of Engel I vs Engel II–IV patients with TLE is shown using an ROC curve. The blue curve was calculated using all patients with TLE, whereas the orange curve was calculated using only patients with TLE who were negative for HS on their MRI. The black dashed line depicts random chance. dFC, dynamic functional connectivity; HS, hippocampal sclerosis; MRI, magnetic resonance imaging; TLE, temporal lobe epilepsy; SAH, selective amygdalohippocampectomy.

We next quantified the dFC peak index in the resections of patients and related this to seizure outcomes at both 1 and 3 years after surgery. Examples of two patients who received SAH surgeries are shown in Figure 4A. Patient C had high dFC peak index in their resected medial temporal lobe and was seizure‐free 3 years after surgery, whereas Patient D did not have increased dFC peak index in their resected medial temporal lobe and experienced seizure recurrence after surgery (Figure 4A). dFC maps of the patients with frontal and parietal lobe epilepsy are shown in Figure 4B. Patient E had a frontal lobe resection that aligned with high dFC peak index and was Engel class I at 3 years after surgery. Patient F had a parietal lobe resection that was between regions of slightly elevated and low dFC peak index and remained Engel I at 3 years after surgery (Figure 4B). Across the cohort, there was no difference in the resected dFC peak index between patients with TLE and Engel I vs Engel II–IV outcomes 1 year after surgery (AUC = .59, 95% CI .38–.76, *p* = .3; Figure S2). However, patients with Engel I outcomes 3 years after surgery had higher dFC peak index in their resection than patients with Engel II–IV outcomes, with moderate distinguishability between the groups (AUC = .80, 95% CI .62–.90, *p* = .0002; Figure 4C,D, Table S3), even in patients who were HS‐negative on MRI (AUC = .79, 95% CI .47–.95, *p* = .04; Figure 4C,D, Table S4).

### 3.5 Clinical correlates of dFC peak index

**FIGURE 5:**
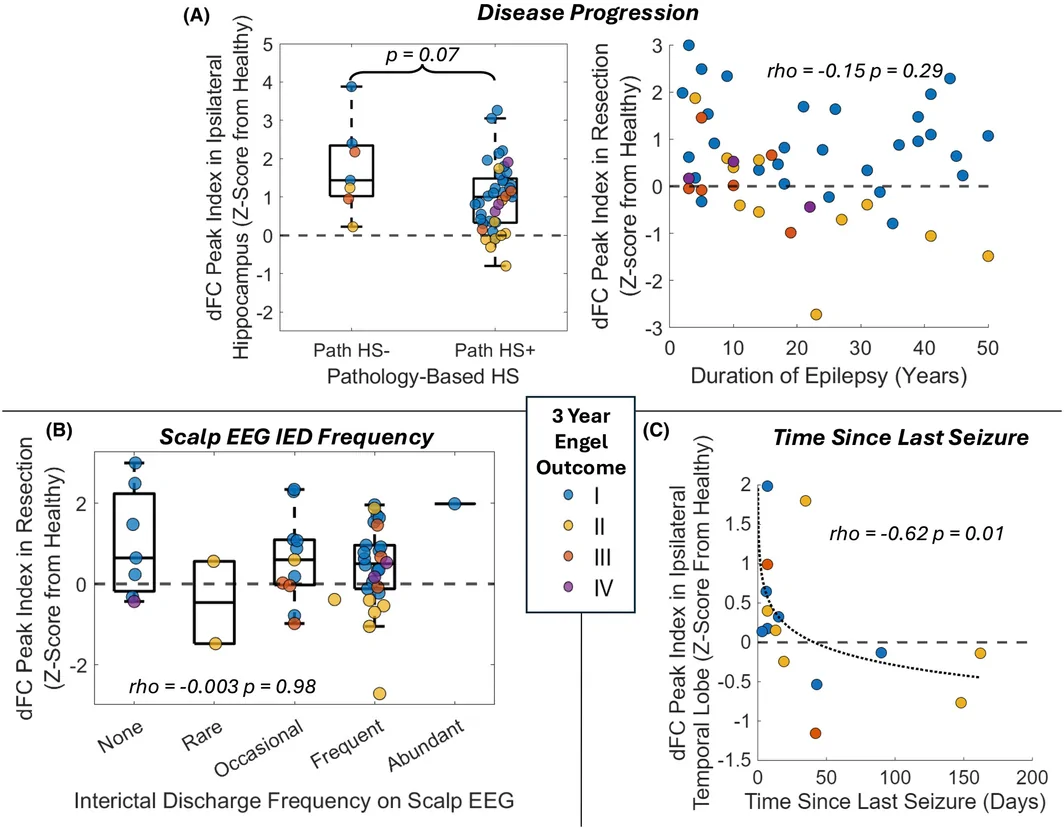
Comparison of dFC peak index to clinical measures. (A) Relationship of dFC peak index to measures of disease progression. Comparisons of dFC peak index within the ipsilateral (resection‐side) hippocampus to HS determined by postoperative pathology (left) and dFC peak index within the resection to duration of epilepsy (right). (B) Relationship between dFC peak index in the resection and interictal epileptic discharge frequencies reported from inpatient scalp EEG recordings. (C) Comparison of dFC peak index within the ipsilateral temporal lobe to time since last seizure. Note that only a subset of patients (*n* = 15) had time since last seizure recorded. The dotted line is a trendline for visualization purposes only. Datapoints are colored by patients' 3‐year Engel outcome. The black dashed line represents the healthy control mean in all plots. dFC, dynamic functional connectivity; EEG, electroencephalography; HS, hippocampal sclerosis; IED, interictal epileptic discharge.

We next tested whether dFC peak index changed with disease progression. We found that dFC peak index trended lower in sclerotic hippocampi as confirmed by postoperative pathology (Cliff's *δ* .42, 95% CI −.14 to .76, *p* = .07; Figure 5A, left) but was not correlated with duration of epilepsy (rho = −.15, 95% CI −.44 to .16, *p* = .29; Figure 5A, right), age at onset (rho = .04, 95% CI −.25 to .33, *p* = .78), age (rho = .01; 95% CI −.28 to .30, *p* = .93), or sex (Cliff's *δ* .19, 95% CI −.22 to .50, *p* = .27). We next evaluated whether dFC peak index was directly related to interictal epileptic discharge‐related measures: interictal discharge frequencies from inpatient scalp EEG studies and time since the last seizure (which is known to temporally modulate interictal discharge frequency^31^). dFC peak index within the resection was not related to interictal discharge frequencies from scalp EEG (rho −.003, 95% CI −.32 to .30, *p* = .98; Figure 5B). In the subset of patients with time since last seizure recorded (*n* = 15), time since last seizure had a negative trend with dFC peak index in the resection (rho = −.42, 95% CI −.77 to .13, *p* = .12) but had a significant negative correlation with dFC peak index of the entire temporal lobe ipsilateral to the resection (rho = −.62, 95% CI −.85 to −.09, *p* = .01; Figure 5C).

## 4 DISCUSSION

In this study we used fMRI to detect negative dFC peaks, moments of connectivity change that relate to interictal epileptic activity in a cohort primarily composed of patients with mesial TLE. This method is grounded in the hypothesis that interictal epileptic discharges lead to fMRI activations in the EZ and deactivations in the DMN, resulting in negative dFC peaks between these regions. Patients with TLE had elevated rates of negative dFC peaks in their epileptic medial temporal lobes compared to both healthy controls and patients' contralateral medial temporal lobes. Resection of regions with increased rates of dFC peaks was related to better seizure outcomes. These results suggest that epileptic tissue exhibits abnormal transient connectivity disruptions with the DMN, potentially reflecting interictal epileptic activity, and that detection of these disruptions could aid in surgical planning.

We leveraged the edge timeseries method^21^ to detect negative dFC peaks, which we hypothesized to reflect interictal epileptic activity, similar to previous edge timeseries studies that detected changes in dFC related to external stimuli.^21^, ^32^ Prior simultaneous EEG–fMRI work has also shown that interictal discharges can transiently reduce dFC between the EZ and DMN,^33^ supporting this hypothesized relationship. Although we found that negative dFC peaks were present in healthy controls, which likely represent normal activity fluctuations, patients had higher rates of these peaks in epileptic regions. We additionally found that patients had decreased dFC peak index in their ipsilateral medial precentral gyrus and the contralateral operculum. Although decreases in dFC peak index are less straightforward to interpret than increases, they may reflect abnormal effects to healthy brain activity patterns in the broader network, rather than effects directly to IEDs.

Patients with unilateral TLE had higher dFC peak index in their epileptic vs contralateral hippocampi, whereas controls had no difference between their hippocampi. dFC peak index laterality could separate right from left TLE very well (AUC = .94), even in patients who were HS‐negative on MRI (AUC = .96). This indicates that the dFC peak index may provide useful lateralizing information in cases that cannot be lateralized with conventional clinical imaging. dFC peak index laterality was not different between bilateral TLE and controls, as expected, since both hippocampi are presumed to be epileptic in these patients.

Resection of tissue with higher dFC peak index was related to better 3‐year seizure outcomes after surgery in TLE. dFC peak index in the resection could separate patients with good (Engel I) from worse (Engel II–IV) outcomes moderately (AUC = .80), even in cases that were HS‐negative on MRI (AUC = .79). This supports the hypothesis that the dFC peak index may be an indicator of epileptic tissue, since patients with good outcomes likely had their true EZ resected. However, resection of low dFC peak index in patients with worse outcomes could reflect mis‐localization of the EZ, resection of a less epileptogenic focus in multifocal epilepsy, or an epileptogenic network that is not amenable to surgery. The two patients with frontal and parietal resections had Engel I outcomes and elevated dFC peak index within their resections, suggesting applicability of the dFC peak index beyond TLE; however, a larger cohort of extratemporal patients would be needed to verify this. These data suggest that the dFC peak index may provide localizing information beyond standard preoperative assessments, given that the measure distinguished patients who responded well to surgery from those who did not.

In the subset of patients who had time since last seizure recorded, dFC peak index was higher in the ipsilateral temporal lobe of those who recently had a seizure but quickly decreased to healthy control levels in patients who had not had a seizure in over ~7 days. This corresponds with previously observed increases in interictal epileptic discharge frequency following seizures^31^, ^34^ and suggests that scanning soon after a seizure may improve dFC peak detection.

dFC peak index was not correlated with scalp EEG interictal discharge frequency, which was unexpected. However, interictal discharge frequency can vary significantly over time^35^, ^36^ and may differ between medication states during the scalp EEG recording (medication tapering) and the MRI scan (prescribed dosage). Scalp EEG also underestimates interictal discharges, particularly from deep sources such as the hippocampus.^37^, ^38^ This suggests that detection of dFC peaks could be valuable in patients who are ineligible for simultaneous EEG‐fMRI due to very infrequent or unidentifiable interictal epileptic discharges on scalp EEG.

Previous studies have aimed to localize interictal epileptic activity using fMRI without simultaneous EEG by detecting fMRI signal increases,^17^, ^18^, ^19^ yet, distinguishing these increases from background oscillations in the fMRI signal is challenging. Thus, here we included the criterion of negative dFC in addition to fMRI signal increases to improve the specificity in detecting interictal activity. Methods aimed at localizing interictal epileptic activity without the need for manual identification of interictal discharges on EEG^39^, ^40^, ^41^ have also been developed, but still require simultaneous EEG recordings. Several studies have suggested that independent component analysis can detect interictal epileptic activity in fMRI without simultaneous EEG,^42^ although identifying which components reflect epileptic activity remains largely subjective. Other approaches, such as static FC^43^, ^44^ and normative modeling of both fMRI metrics^45^ and timeseries,^46^ have been proposed to localize or lateralize the EZ without EEG. However, unlike the dFC peak index, these methods are not specifically designed to capture interictal discharge–like activity.

There are several limitations to this study. First, we presented examples of negative dFC peaks following interictal discharges but did not statistically test this relationship, since our main cohort only had fMRI without simultaneous EEG collected. Future simultaneous EEG‐fMRI studies can test whether negative dFC peaks are associated with interictal discharges or other interictal activity, such as high‐frequency oscillations^47^ and intermittent delta activity.^48^ We designed dFC peak index to generalize across focal epilepsies and tested the method in two patients with extra‐TLE; however, our primary analysis was in patients with TLE. Thus further studies are needed to validate this generalization. The dFC peak index also assumes both fMRI activation in the EZ and deactivation in the DMN in response to interictal discharges; however, occasional cases of interictal discharge‐induced deactivations in the EZ have been reported.^6^ Moreover, the DMN comprises several regions that can be differentially deactivated depending on the location of the discharge,^12^, ^13^, ^15^, ^49^, ^50^ which can affect the generalization of dFC peak index to various focal epilepsies. We used the entire DMN in this study to avoid tailoring the method to specific discharge locations, but more nuanced approaches could be developed for different epilepsy subtypes. Interictal discharges also occur more frequently in lower arousal states,^51^ which could be utilized to improve the detection of interictal activity with the dFC peak index. However, arousal may also introduce unwanted variability into the dFC peak index, since it can influence healthy fMRI activity and connectivity.^52^, ^53^, ^54^ Furthermore, we used fMRI data with a temporal resolution of 2 s in this work, whereas interictal epileptic discharges often occur on the sub‐second scale. Finally, high dFC peak index likely indicates an area of dysfunction that is broader than the EZ (i.e., the irritative zone), which could be used in conjunction with other modalities to localize the EZ more accurately.^55^

We developed the dFC peak index, a method aimed at detecting and localizing interictal epileptic activity using resting fMRI without simultaneous EEG. Our results indicate that the dFC peak index is elevated in epileptic tissue in TLE, suggesting that this method may detect interictal epileptic activity with the potential to aid in surgical guidance. Future work can improve this method with faster fMRI protocols and more sophisticated models of how interictal activity impacts FC dynamics across various epilepsy etiologies.

## ETHICAL PUBLICATION STATEMENT

We confirm that we have read the Journal's position on issues involved in ethical publication and affirm that this report is consistent with those guidelines.

## AUTHOR CONTRIBUTIONS

L.E.S. and V.L.M. contributed to the conception and design of the study. L.E.S., A.R., G.S.M., B.P.R., S.M., A.E.V., D.J.E., and V.L.M. contributed to the acquisition and analysis of the data. L.E.S., G.S.M., S.W.R., S.M., A.E.V., C.C., D.J.E., and V.L.M. contributed to the interpretation of data and drafting of the manuscript.

## FUNDING INFORMATION

This study was funded by the National Institute for Neurological Disorders and Stroke at the National Institutes of Health: F31 NS135908, R01 NS075270, R01 NS108445, R01 NS110130, and R00 NS097618. Simultaneous EEG‐fMRI data collection was supported by: Emilia‐Romagna—SSN young researcher grant (Project Code GR‐2013‐02358466); SSN—RF grant: (Project Code NET‐ 2013‐02355313); Emilia‐Romagna regional funding to the Azienda Ospedaliera‐Universitaria di Modena “Centro hub chirurgia epilessia” (DGR 1172/18); Dipartimento di eccellenza ‘2018–2022’, MIUR, Italy, to the Department of Biomedical, Metabolic and Neural Sciences.

## CONFLICT OF INTEREST STATEMENT

None of the authors have any conflicts of interest to disclose.

## CONSENT

This study was approved by the Vanderbilt University Institutional Review Board and informed consent was obtained from all participants.

## Supporting information


**Figure S1.** Regional group differences in dynamic functional connectivity (dFC) peak index between patients with unilateral temporal lobe epilepsy (TLE) and healthy controls. Regions with a significant difference in dFC peak index between groups are shown in blue, denoting regions in which patients with TLE have fewer peaks than controls, and red, denoting regions in which patients with TLE have a higher number of peaks than controls (*p*_FWE_ <.05). Ipsi, ipsilateral to epileptogenic zone; Contra, contralateral to epileptogenic zone.
**Table S1.** Confusion matrix for optimal operating point to separate right from left temporal lobe epilepsy with dynamic functional connectivity (dFC) peak index laterality in the hippocampus.
**Table S2.** Confusion matrix for optimal operating point to separate *hippocampal sclerosis (HS)‐negative* right from left temporal lobe epilepsy with dynamic functional connectivity (dFC) peak index laterality in the hippocampus.
**Table S3.** Confusion matrix for optimal operating point to separate Engel class I from Engel II–IV 3‐Year seizure outcomes with dynamic functional connectivity (dFC) peak index in the resection.
**Table S4.** Confusion matrix for optimal operating point to separate *hippocampal sclerosis (HS)‐negative* Engel I from Engel II–IV 3‐Year seizure outcomes in with dynamic functional connectivity (dFC) peak index in the resection.
**Figure S2:** Surgical resection of high dynamic functional connectivity (dFC) peak index does not correlate with 1‐year seizure outcomes. (A) dFC peak index in the resection vs seizure outcomes 1 year after surgery. Blue datapoints depict patients with temporal lobe epilepsy (TLE) who were hippocampal sclerosis (HS)‐positive on MRI and red datapoints depict patients with TLE who were HS‐negative on MRI. The black dashed line represents the healthy control mean. (B) Separation of Engel class I vs Engel II–IV patients with TLE is shown using an receiver‐operating characteristic (ROC) curve. The blue curve was calculated using all patients with TLE, whereas the orange curve was calculated using only patients with TLE who were negative for HS on their MRI. The black dashed line depicts random chance. HS, hippocampal sclerosis.

## Data Availability

The imaging data that support the findings of this study are available on Pennsieve (https://doi.org/10.26275/vvq3‐pdhe and https://doi.org/10.26275/e22w‐ksut). Code and data for the analysis and dFC peak index method are available at https://github.com/vmorgan‐lab/dfc‐peak‐index.
