# Supplementary material for: The dynamic functional connectivity peak index: Detection of interictal epileptic activity with fMRI
Source: Epilepsia. 2026 Jun 11;67(9):4702–16. doi: 10.1002/epi.70335 (PMC13590121; doi:10.1002/epi.70335)
Supplement: Supplementary file 1 [file EPI-67-4702-s001.docx]

**Supplementary Information for:**

**The dynamic functional connectivity peak index: detection of interictal epileptic activity with fMRI**

Lucas E. Sainburg,^1,2^ Alexandra Roche,^2^ Ghassan S. Makhoul,^1,2^ Baxter P. Rogers,^1,2^ Shawniqua Williams Roberson,^1,3^ Stefano Meletti,^4,5^ Anna E. Vaudano,^4,5^ Catie Chang,^1,2,6^ Dario J. Englot,^1,2,3,6,7^ and Victoria L. Morgan^1,2,3,7^

**Author affiliations:**

1 Department of Biomedical Engineering, Vanderbilt University, Nashville, TN, USA

2 Vanderbilt University Institute of Imaging Science, Vanderbilt University Medical Center, Nashville, TN, USA

3 Department of Neurology, Vanderbilt University Medical Center, Nashville, TN, USA

4 Department of Biomedical, Metabolic and Neural Science, University of Modena and Reggio Emilia, Modena, Italy

5 Neurophysiology Unit and Epilepsy Center, Neuroscience Department, Modena AOU, Modena, Italy

6 Department of Electrical and Computer Engineering, Vanderbilt University, Nashville, TN, USA

7 Department of Neurological Surgery, Vanderbilt University Medical Center, Nashville, TN, USA

**Supplemental Methods for Part 1: Illustration of Negative dFC Peaks**

*Simultaneous EEG-fMRI Acquisition and Preprocessing*

Simultaneous EEG-fMRI data from two patients were included for the purpose of presenting examples of negative dynamic functional connectivity (dFC) peaks after interictal epileptic discharges. Patient 1 (39-year-old female) was diagnosed with left medial temporal lobe epilepsy (TLE), while Patient 2 (19-year-old male) was diagnosed right lateral TLE. Both patients underwent simultaneous EEG-fMRI at the University of Modena and Reggio Emilia.

The MRI data were acquired on a Philips 3 T MRI and included a T1-weighted scan for anatomy (1 mm isotropic) and a T2*-weighted eyes-closed resting-state fMRI scan (Patient 1: 3 × 3 × 4 mm, repetition time = 2 s, echo time = 35 ms, 8 min; Patient 2: 3.6 × 3.6 × 4 mm, repetition time = 3 s, echo time = 50 ms, 10 min).

The fMRI data were preprocessed with SPM12 (https://www.fil.ion.ucl.ac.uk/spm/software/spm12/), which included slice timing correction, motion correction, normalization to Montreal Neurological Institute (MNI) coordinates and spatial smoothing with a 6 mm full width at half-maximum Gaussian kernel. RETROICOR^1^ was not performed since cardiac and respiratory data were not recorded.

EEG data were collected with a Micromed 32-channel MR-compatible EEG system (Mogliano Veneto, Italy) at a sampling rate of 1024 Hz. EEG data underwent gradient, ballistocardiogram artifact removal and independent component analysis^2^ and an experienced electroencephalographer (AEV) manually detected interictal epileptic discharges on the preprocessed EEG.

*Interictal Epileptic Discharge Activation Maps*

Interictal epileptic discharge activation maps were obtained using SPM12. The timings of the interictal epileptic discharges were convolved with the canonical hemodynamic response function in SPM12 and the resulting timeseries were used as a regressor along with the six translational and rotational motion parameters. Activation maps were generated for the interictal epileptic discharge regressor using an uncorrected threshold of p < 0.001 and cluster size of > 10 voxels.

*Regional Timeseries and dFC Calculation*

To calculate the regional timeseries and dFC, the six motion parameters as well as the mean cerebrospinal fluid and white matter signals were first regressed out of the fMRI data. The fMRI data were then bandpass filtered between 0.0083-0.1 Hz. Preprocessed timeseries were then extracted from both the default mode network (DMN) and the largest activated cluster from the interictal discharge activation map. The fMRI timeseries from these regions were temporally Z-scored by de-meaning and dividing by their temporal standard deviation. These Z-scored fMRI timeseries were then multiplied together element-wise to obtain dFC, following the edge timeseries approach^3^.

**Supplementary Results**

Supplementary Figure 1: Regional group differences in dFC peak index between patients with unilateral TLE and healthy controls. Regions with a significant difference in dFC peak index between groups are shown with blue denoting regions in which patients with TLE have fewer peaks than controls, and red denoting regions in which patients with TLE have a higher number of peaks than controls (p_FWE_ < 0.05). Ipsi: ipsilateral to epileptogenic zone; Contra: contralateral to epileptogenic zone.

Supplementary Table 1: Confusion Matrix for Optimal Operating Point to Separate Right from Left TLE with dFC Peak Index Laterality in the Hippocampus

|  | Predicted Left TLE | Predicted Right TLE |
| --- | --- | --- |
| True Left TLE | 14 | 2 |
| True Right TLE | 3 | 30 |

Optimal operating point was determined using the Youden Index.

Supplementary Table 2: Confusion Matrix for Optimal Operating Point to Separate *HS-Negative* Right from Left TLE with dFC Peak Index Laterality in the Hippocampus

|  | Predicted Left TLE | Predicted Right TLE |
| --- | --- | --- |
| True Left TLE | 4 | 0 |
| True Right TLE | 2 | 12 |

Optimal operating point was determined using the Youden Index.

Supplementary Table 3: Confusion Matrix for Optimal Operating Point to Separate Engel I from Engel II-IV 3-Year Seizure Outcomes with dFC Peak Index in the Resection

|  | Predicted Engel I | Predicted Engel II-IV |
| --- | --- | --- |
| True Engel I | 19 | 10 |
| True Engel II-IV | 3 | 17 |

Optimal operating point was determined using the Youden Index.

Supplementary Table 4: Confusion Matrix for Optimal Operating Point to Separate *HS-Negative* Engel I from Engel II-IV 3-Year Seizure Outcomes in with dFC Peak Index in the Resection

|  | Predicted Engel I | Predicted Engel II-IV |
| --- | --- | --- |
| True Engel I | 7 | 3 |
| True Engel II-IV | 1 | 7 |

Optimal operating point was determined using the Youden Index.

Supplementary Figure 2: Surgical resection of high dFC peak index does not correlate with 1-year seizure outcomes. (A) dFC peak index in the resection versus seizure outcomes 1 year after surgery. Blue datapoints depict patients with TLE who were HS-positive on MRI and red datapoints depict patients with TLE who were HS-negative on MRI. The black dashed line represents the healthy control mean. (B) Separation of Engel I vs Engel II-IV patients with TLE is shown using an ROC curve. The blue curve was calculated using all patients with TLE, while the orange curve was calculated using only patients with TLE who were negative for HS on their MRI. The black dashed line depicts random chance. HS: hippocampal sclerosis.

**References**

1.	Glover GH, Li TQ, Ress D. Image-based method for retrospective correction of physiological motion effects in fMRI: RETROICOR. *Magnetic Resonance in Medicine*. 2000;44(1):162-167. doi:10.1002/1522-2594(200007)44:1%3C162::AID-MRM23%3E3.0.CO;2-E

2.	Avanzini P, Vaudano AE, Vignoli A, et al. Low frequency mu-like activity characterizes cortical rhythms in epilepsy due to ring chromosome 20. *Clinical Neurophysiology*. 2014;125(2):239-249. doi:10.1016/j.clinph.2013.07.009

3.	Esfahlani FZ, Jo Y, Faskowitz J, et al. High-amplitude cofluctuations in cortical activity drive functional connectivity. *PNAS*. 2020;117(45):28393-28401. doi:10.1073/pnas.2005531117
